# Subjective and objective observation of skin graft recovery on Indonesian local cat with different periods of transplantation time

**DOI:** 10.14202/vetworld.2016.481-486

**Published:** 2016-05-15

**Authors:** Erwin, Gunanti, Ekowati Handharyani, Deni Noviana

**Affiliations:** 1 Laboratory of Clinic and Surgery, Faculty of Veterinary Medicine, Syiah Kuala University, Banda Aceh, Indonesia; 2 Department of Clinic Reproduction and Pathology, Faculty of Veterinary Medicine, Bogor Agricultural University, Bogor, Indonesia

**Keywords:** auto skin graft, cats, observation

## Abstract

**Aim:**

The success of a skin graft in a cat is highly dependent on the granulation formed by the base of recipient bed. Granulation by the base of recipient bed will form after several days after injury. This research aimed to observe subjective and objective profile of skin graft recovery on forelimb of cats with different periods of donor skin placement.

**Materials and Methods:**

Nine male Indonesian local cats aged 1-2 years old, weighing 3-4 kg were divided into three groups. The first surgery for creating defect wound of 2 cm×2 cm in size was performed in the whole group. The wound was left for several days with the respective interval for each group, respectively: Group I (for 2 days), Group II (for 4 days), and Group III (for 6 days). In the whole group, the second surgery was done by the harvesting skin of thoracic area which then applied on recipient bed of respective groups.

**Result:**

The donor skin on Group II was accepted faster compared to Group I and Group III. The donor skin did not show color differences compared to surrounding skin, painless, bright red in bleeding test had faster both hair growth and drug absorption. Test toward the size of donor skin and the effect of drugs did not show a significant difference between each group.

**Conclusion:**

The observe subjective and objective profile of skin graft recovery on forelimb of cats on Group II were accepted faster compared to Group I and III.

## Introduction

The advancement of oncology surgery to treat skin defects caused by various traumas indicated that skin reconstruction surgery in dogs and cats is developing fast [1]. Skin defects on the limb of cats are a challenge for a veterinarian to improve the reconstruction technique. The skin graft on the cat’s limb requires high precision which is the wound base should provide adequate space for donor skin placement. Before skin graft surgery was conducted, the base of recipient bed must be left out for granulation to form and vascularization preserved. A proper and precise planning and non-traumatic surgery technique are a must to minimize wound tissue distention [2,3].

The skin graft is harvesting skin from donor’s body, during which donor skin will loose blood supply as it was relocated to recipient bed. Anaerobic respiration occurred when donor skin does not receive blood supply. Wound management by skin graft could be used with several options of techniques [3,4]. Skin graft consists of autograft, isograft, allograft, and xenograft. For cat and dog, both autograft and isograft are more often skin graft surgery used to treat clinical cases. Two type of auto skin graft techniques most frequent used are full thickness skin graft and partial thickness skin graft [2,5].

The success of skin graft greatly relies on good recipient bed preparation so that donor skin and recipient skin could attach. The proper base of wound preparation took several days indicated by reddening by the wound’s surface. Clinically, the skin graft is considered a success when donor skin is received well and can merge with recipient skin [6]. Subjectively, the success of skin graft in dogs can be determined by several tests such as observation skin color, bleeding, hair growth, and pain response. The objective evaluation could be observed by the ability to absorb drug through donor skin and by the manifestation of the drug’s effect [7,8]. This research aimed to observe the success of skin graft on the forelimb of Indonesian local cats clinically via subjective and objective observation with different periods of donor skin placement.

## Materials and Methods

### Ethical approval

This research has been approved by the Animal Care and Use Committee of Veterinary Teaching Hospital, Faculty of Veterinary Medicine, Bogor Agricultural University (IPB) with approval number: 19-2014 IPB.

### Research procedure

This research used nine male of Indonesian local cats aged 1-2 years old, weighing 3-4 kg. The cats were adapted to individual cages for 1 month and fed 3 times daily with water *ad libitum*. The cats were given amoxicillin and clavulanic acid antibiotics (62.5 mg/kg body weight [BW]) and metronidazole (17 mg/kg BW), praziquantel and pyrantel embonate (5 mg/kg BW), and vitamin supplements as acclimatization drugs 1-month period to surgery. The cats were bathed and fasted for 8 h before surgery to prevent vomiting. They were given atropine sulfate 0.25% (0.04 mg/kg BW) as premedication and combination of ketamine 10% (10 mg/kg BW) and xylazine (1 mg/kg BW) as general anesthetic [9]. The first surgery was preceded by shaving hair and disinfecting lateral area of the forelimb. It was then followed by making an incision wound defect 2 cm×2 cm. The wound was wrapped by sterile gauze added by povidone iodine then left for several days, differing between treatment groups: Group I for 2 days, Group II for 4 days, and Group III for 6 days. Second surgery was done by harvesting skin by the thoracic area and applied it on the base of recipient bed, which previously has been cleaned of subcutaneous tissue [10]. Finally, the donor skin and recipient skin were stitched by a simple interrupted suture with polypropylene 3.0 USP thread. Skin graft area was wrapped by framycetin sulfate which was replaced on day 3, 6, 9, 12, and 18. For maintenance, amoxicillin and clavulanic acid 62.5 mg/kg BW and flunixin meglumine 1 mg/kg BW were given 2 times daily for 8 days. Clinical assessment of skin graft recovery was determined by subjective and objective clinical parameter observation [7,11,12].

### Subjective parameters

They are as follows:

- Donor skin color changes were observed on day 3, 6, 9, 12, and 18 after skin graft surgery. The color changes of donor skin are scored by the category: 4 (black/necrosis), 3 (ischemia), 2 (hyperemia), and 1 (same as surrounding skin).
- Pain response test was done on day 3, 6, 9, 12, and 18 after skin graft surgery by applying pressure on donor skin site and observing cat’s reflex reaction under the scoring: 3 (no pain/necrosis), 2 (pain), and 1 (no pain/healed).
- Donor skin size was observed on day 3, 6, 9, 12, and 18 after skin graft surgery measured by length times width (cm).
- Bleeding test was done on day 18 after skin graft surgery by making incision (1 mm) on donor skin and observing the blood quality. The exiting blood quality is scored under the category: 2 (blood is bright red and takes a long time to appear after incision was made) and 1 (blood is bright red and appearing right after incision was made).
- Observation of hair growth by observing the growing of hair on donor skin of each cat.

### Objective parameters

They are as follows:

- Drug absorption ability test was done on day 18 after skin graft surgery by injecting 0.2 mL of NaCl 0.9% under the donor skin and then measuring the absorption time.
- Drug reaction test was done on day 18 after skin graft surgery by injecting 0.2 mL adrenaline under the donor skin and observing the onset for sympathetic nerve response reaction to appear [7].

### Statistical analysis

Quantitative data of subjective and objective observation were analyzed by analysis of variance. All data were processed by the statistical package for social sciences (SPSS) 21 software.

## Results

### Donor skin color change

**Figure-1:**
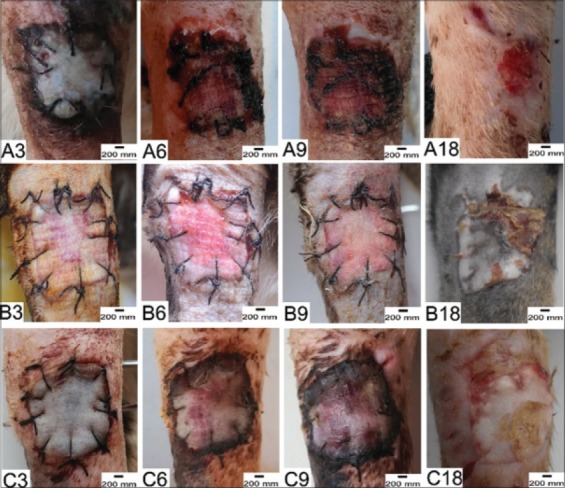
Clinical representation donor’s skin color each group. Group I (A3: Skin graft status after 3 days, A6: Skin graft status after 6 days, A9: Skin graft status after 9 days, and A18: Skin graft status after 18 days), Group II (B3: Skin graft status after 3 days, B6: Skin graft status after 6 days, B9: Skin graft status after 9 days, and B18: Skin graft status after 18 days), and group III (C3: Skin graft status after 3 days, C6: Skin graft status after 6 days, C9: Skin graft status after 9 days, and C18: Skin graft status after 18 days).

Donor skin color observation for the first few days after skin graft surgery showed ischemia on Group I with average scoring 2.60±1.12; hyperemia on Group II with average scoring 1.60±0.83; hyperemia on Group III with average scoring 1.87±0.84. Donor skin color change scoring for each observation time is shown in Table-1. On day 6, after skin graft surgery, donor skin color on Group I still appeared ischemia, whereas Group II and Group III already appeared hyperemia. Day 9 after skin graft surgery, Group I donor skin had not merged with recipient skin and showed hyperemia, whereas Group II had the edges of its donor skin starting to merge with recipient skin while Group III had the donor skin appeared hyperemia but had not merged completely with recipient skin. On day 12, donor skin of Group II had started to be received and marked by the merging of the edges of the wound of recipient skin. However, in Group I, only part of the edges of donor skin merged, with one cat showed rejection in the form of necrosis of donor skin. On day 18, after skin graft surgery, all donor skin had merged well, and flaking skin had peeled off. Color changes of the donor skin of each group are represented on Figure-1.

**Table-1:** Observation of subjective donor skin color changes.

Observation days	Treatment group (scoring±SD)		

	I	II	III
3	3.0±0.0^a+^	3.0±0.0^a^*	3.0±0.0^a^*
6	3.0±1.0^a+^	2.0±0.0^b^*	2.3±0.6^b^*
9	2.7±1.2^a+^	1.0±0.0^cd^*	1.7±0.6^c^*
12	2.3±1.5^a+^	1.0±0.0^cd^*	1.3±0.6^c^*
18	2.0±1.7^a+^	1.0±0.0^cd^*	1.0±0.0^c^*

Value with different superscripts in the same column (a-d) and row (^+*^) indicate significant difference (p<0.05). The changes skin color given a categorized scoring; 4 (necrosis), 3 (ischemia), 2 (hyperemia), 1 (same as surrounding skin). SD=Standard deviation

### Pain response test

Day 3 after skin graft surgery, pain response test showed that all cat gave pain response. Pain response started to diminish on day 6, 9, and 12 on Group II, whereas for Group I and III the pain response started to diminish from day 9 and 12. Table-2 presented the average result of pain response test in each group and each observation time. In the first days, after skin graft surgery, temporary pain response is very desirable. The presence of pain is one of inflammation symptoms. Inflammation phase happened on 24-48 h after skin graft surgery was conducted. Group II cats showed pain response up to day 6; and on day 9; pain response had decreased and showing that donor skin had been received well by the recipient. Cats of Group I and III still showed pain response as a result of donor skin not yet being well received by the recipient.

**Table-2:** Observation of subjective pain response test.

Observation days	Treatment group (scoring±SD)		

	I	II	III
3	2.0±0.0^a+^	2.0±0.0^a^*	2.0±0.0^a^*
6	2.0±0.0^a+^	2.0±0.0^a^*	2.0±0.6^a^*
9	2.0±0.6^a+^	1.3±0.6^b^*	2.0±0.0^a^*
12	2.0±1.0^a+^	1.0±0.0^b^*	1.3±0.6^b^*
18	1.6±1.2^a+^	1.0±0.0^b^*	1.0±0.0^b^*

Value with different superscripts in the same column (a-b) and row (^+*^) indicate significant difference (a-e, p<0.05). The pain test given a categorized scoring; 3 (no pain/necrosis), 2 (pain) and 1 (no pain/healed). SD=Standard deviation

### Donor skin size

The donor skin size of Group I, II, and III showed the tendency of shrinking, shown in Table-3. The shrinking size of donor skin ranged between 0.2 and 0.5 cm, with differences in each observation time. On Group II, donor skin size was shrunk relatively less than Group III, with Group I showing the most shrinkage. Although statistically no significant difference was found between three treatment groups, clinically donor skin in Group I was shrunk more than the other groups. It was caused by the poor condition recipient bed, which resulted in necrosis in parts of the donor skin.

**Table-3:** Observation of subjective donor skin size.

Observation days	Treatment group (cm±SD)		

	I	II	III
3	4.0±0.0^a^*	4.0±0.0^a^*	4.0±0.0^a^*
6	4.0±0.0^a^*	3.7±0.1^b^*	3.8±0.0^b^*
9	3.7±0.3^a^*	3.7±0.1^c^*	3.5±0.1^c^*
12	2.3±1.9^a^*	3.5±0.1^d^*	3.4±0.1^d^*
18	2.2±1.9^a^*	3.5±0.1^d^*	3.3±0.1^d^*

Value with different superscripts in the same column (a-d) and row (^+*^) indicate significant difference (a-e, p<0.05). SD=Standard deviation

### Bleeding test

Bleeding test was done on day 18 after skin graft surgery. Blood that came out of bleeding test in Group II was bright red and came out quickly after incision. In Group III, the blood was also bright red even though it took a longer time to come out after incision compared to Group II. Bleeding test in Group I showed bright red blood as well and took a longer time to come out compared to Group II and III. Table-4 presented the average result of bleeding test on each group and each observation. Bleeding test of Group II and III showed better result, marked by fast bleeding and with the bright red blood that came out being the same as the blood in other healthy are of the body. On Group, I, the time needed for blood to come out after the incision was relatively longer with rather pinkish caused the formation of connective tissue.

**Table-4:** Observation of subjective bleeding test.

Observation days	Treatment group (scoring±SD)		

	I	II	III
18	2.0±0.6^+^	1.0±0.0*	1.3±0.6*

Value with different superscripts in the same line indicates significant difference (^+*^, p<0.05). The bleeding test given a categorized scoring; 2 (blood is bright red and takes a long time to appear after incision was made), 1 (blood is bright red and appearing right after incision was made). SD=Standard deviation

### Observation of hair growth

Hair grew earlier in Group II was occurred than Group I and III. The average time for hair to grow in each group is presented in Table-5. Based on clinical representation, donor skin of Group II was completely received by the recipient, which allowed hair to grow early. Several epidermis stratum of donor skin in Group III shed, which resulted in disturbance of hair growth. The same occurrence was found on Group I. Growth of dense connective tissue by hypodermal layer would delay Group I cat hair to grow.

**Table-5:** Observation of subjective hair growth.

Observation days	Treatment group (day±SD)		

	I	II	III
	25.0±1.4^+^	18.7±1.2*	26.7±1.2*

Value with different superscripts in the same line indicates significant difference (^+*^, p<0.05). SD=Standard deviation

### Absorption and reaction drugs test

Objective test consists of drug absorption test and subcutaneously administered drug’s effect by the donor skin. An amount of 0.2 mL NaCl 0.9% solution was administered below the skin. Absorption time of the solution was measured. Absorption time was faster in Group II compared to Group I and III was presented on Table-6, whereas the onset of drug effect did not show a significant difference between each treatment group was presented on Table-7. In the other hand, 0.2 mL sympathomimetic drugs, i.e. adrenaline was given under the donor skin to observe its ability in absorbing drug, which would then take effect in the form of pupil dilatation. There was no significant difference on sympathomimetic drug effect between each group.

**Table-6:** Observation of objective drug absorption.

Observation time	Treatment group (minute±SD)		

	I	II	III
Min	3.6±0.7^+^	3.1±0.8*	3.3±0.2*

Value with different superscripts in the same line indicates significant difference (^+*^, p<0.05). SD=Standard deviation

**Table-7:** Observation of objective drug reaction.

Observation time	Treatment group (minute±SD)		

	I	II	III
Min	4.1±0.1^+^	4.1±0.1^+^	4.2±0.1^+^

Value with different superscripts in the same line indicates significant difference (^+*^, p<0.05). SD=Standard deviation

## Discussion

The success of skin graft is determined by how donor skin is received by the recipient clinically. Based on subjective and objective observation, skin graft rate of success on Indonesian local cat was as high as 80-90%. Auto skin graft success highly relies on proper recipient bed maintenance, animal treatment for the first few days after surgery, bandage replacement period, and drug usage [13]. Neovascularization between donor and recipient bed was determined by angiogenesis process happening during granulation. Blood vessel formation involves vascular endothelial growth factor through these following phases: Vasodilatation, base membrane dilatation, endothelial cell migration, and endothelial cell proliferation [14].

Skin graft recovery begins by swelling or inflammation that happened 48-72 h after surgery. Swelling or inflammation is caused by inhibition process of hemoglobin degradation products. Swelling decreasement is an indicator of neovascularization occurring between donor skin and recipient bed [10,15]. The skin graft is considered unsuccessful if within 3-5 days after surgery, donor skin appeared cyanotic or black (necrosis). Shedding happened to epidermis layer was occurred in Group III. Sometimes donor skin loses its epidermal layer on the 1^st^ week, appearing like a failed skin graft [3,7]. This can be puzzling, however, generally dermal graft will appear on 2^nd^ week, and donor skin will grow satisfactorily in covering the skin defect. Donor skin color is expected to be the same as surrounding skin. Reddening on donor skin indicated that neovascularization formed between donor skin and recipient bed, which allows donor skin to be accepted by recipient’s body. Hyperemia rate of donor skin was also determined by a good surface of wound condition before skin graft was conducted. An inadequate recipient bed would result in donor skin taking longer time to be accepted by the recipient, or even rejected that is indicated by cyanotic color on donor skin caused by lack of neovascularization [13,16].

The entire surface of recipient bed should be prepared for neovascularization. Neovascularization gave nutrition to donor skin. Donor skin’s subcutaneous layer must be cleared for it may hinder imbibition process [3,7,16]. Donor skin that received inadequate vascularization after skin graft must hold out for at least 48 h. After that, donor skin will absorb protein-containing fluid from capillary blood vessel by the bed of the wound (plasmatic imbibition). During this period, donor skin will attach itself to the tissue below it (the base of the recipient bed) and fibrin will act as biological glue. This biological glue provides scaffolding where new capillary blood vessels could grow on, and recipient base wound could start to accept donor skin. Biological glue is preceded by anastomoses to begin forming new circulation, in which the process is called inosculation. Inosculation phase happens 48 h after surgery, although it is also dependent on the condition of the base of the wound. This phase is marked by the formation of new capillary blood vessel by the recipient bed base that will meet or is connected to donor skin. In this phase, blood already starts to circulate the donor skin to provide nutrition. Based on the explanation, a good wound base that gives support in the form of granulation and vascularization to donor skin is the main determinant in skin graft surgery success [3,13,16]. The part of donor skin that does not receive neovascularization and nutrition will experience necrosis. Tissue with necrosis will be eliminated by neutrophil and macrophage. Fibroblast will grow and be the most dominant cell (proliferation phase) which is responsible in collagen formation [15].

The quality of blood is determined by the number of neovascularization forming underneath the donor skin. Better neovascularization will result in bright red blood. In addition, the density of connective tissue underneath the donor skin also influences the time needed for bleeding to occur. Hypodermal or subcutaneous layer consists of loose connective tissue, which contains a lot of elastic fiber [10]. In pathological condition, this layer formed several cavity filled with fluid (edema) or air (emphysema). Cat hair is a part of dermal part of the skin. The time needed for hair growth is influenced by the donor skin harvesting technique and the amount of connective tissue formed. The lack of hair growth on donor skin may be caused by hair follicle damage during donor skin harvesting process [2,16].

The longer drug absorption time of group I and III was caused by the extensive formation of connective tissue between donor skin and recipient bed. In addition, the lack of neovascularization also contributed to absorption, which resulted in the drug staying under the skin longer before being completely absorbed [16]. There are two phases of drug administration: Pharmacokinetic and pharmacodynamic. Pharmacokinetic phase is associated with dosage, administration frequency, and drug concentration within the body. Subcutaneous administration takes longer absorption time compared to intravenous administration. The rate of absorption and distribution in subcutaneous administration depend on vascularization supply in respective tissues. In the capillary section, absorption is eased by endothelial porous with approximately 3 µm radius. If the compound has reached the blood vessel, the test of the distribution is done by blood circulation. Distribution of respective drug compound is dependent on the size of the molecules, plasma protein and tissue protein bond, solubility, and chemical properties [7,17,18]. Full thickness skin graft can be easily applied to cat under the conditions of proper recipient bed and minimize animal movement for the first few days after skin graft surgery. In addition, immunosuppressive drugs are needed to minimize rejection [19]. The result of this research differs with the one on dogs, where autogenous transplantation can be applied on dogs with little rejection, in accordance with the animal’s own immunity [20].

## Conclusion

This study has successfully performed observe subjective and objective profile of skin graft recovery on forelimb of cats. The recommendation of the best application day of donor skin on recipient bed in descending order is: Group II (very recommended), Group III (recommended), and Group I (not recommended). The study will plausibly be helpful for pet practitioners in Indonesian in dealing with the cats distal limb skin defect.

## Authors’ Contributions

Authors will declare the contribution of each author such as E, G, and DN conceived and designed the anesthesia and surgery. E and EH executed the experiment, analyzed and interpretation of data the tissue. All authors interpreted the critically revised the manuscript for important intellectual contents and approved the final version.

## References

[1] Ferrari R, Boracchi P, Romussi S, Ravasio G, Stefanello D (2015). Application of hyaluronic acid in the healing of non-experimental open wounds:A pilot study on 12 wounds in 10 client-owned dogs. Vet. World.

[2] Degner D.A (2008). Skin graft in dogs and cats.

[3] Nelissen P, White D, Langley-Hobbs S.J, Demetriou J.L, Ladlow J.F (2014). Flaps and graft. Feline Soft Tissue and General Surgery. British Library Cataloguing in Publication Data.

[4] Siegfreed R, Schmokel H, Rytz U, Spreng D, Schawalder P (2004). Treatment of large distal exstremity skin wounds with autogenous full-thickness mesh skin in five cats. Schweiz. Arch. Tierheilkd.

[5] Andreassi A, Bilenchi R, Biagioli M, D’Aniello C (2005). Classification and pathophysiology of skin grafts. Clin Dermatol.

[6] Fowler D (2006). Distal limb and paw injuries. Vet. Clin. North Am. Small Anim. Pract.

[7] Ijaz M.S, Mahmood A.K, Ahmad N, Khan M.A, Farooq U (2012). Viability of split thickness autogenous skin transplantation in canine distal limb reconstruction an experimental evaluation. Pak. Vet. J.

[8] Kreidstein M.L, Pang C.Y, Levine R.H, Knowlton R.J (1991). The isolated perfused human skin flap:Design, perfusion technique, metabolism, and vascular reactivity. Plast Reconstr. Surg.

[9] Tilley L, Smith F (2005). The 5-Minute Veterinary Consult.

[10] Stanley B.J, Pitt K.A, Weder C.D, Fritz M.C, Hauptman J.G, Steficek B.A (2013). Effect of negative pressure wound therapy on healing of free full-thickness skin graft in dog. Vet. Surg.

[11] Bö ttcher P, Zeissler M, Maier J, Grevel V, Oechtering G (2009). Mapping of split-line pattern and cartilage thickness of selected donor and recipient sites for autologous osteochondral transplantation in the canine stifle joint. Vet. Surg.

[12] Nguyen D.Q.A, Potokar T.S, Price P (2010). An objective long-term evaluation of integra (a dermal skin substitute) and split thickness skin grafts, in acute burns and reconstructive surgery. Burns.

[13] Rahal S.C, Mortari A.C, Milton M, Filho M (2007). Mesh skin graft and digital pad transfer to reconstruct the weight-bearing surface in a case report dog. Can. Vet. J.

[14] Bao P, Kodra A, Tomic-Canic M, Golinko M.S, Ehrlich H.P, Brem H (2009). The role of vascular endothelial growth factor in wound healing. J. Surg. Res.

[15] Mathes D.W, Noland M, Graves S, Schlenker R, Miwongtum T, Storb T (2010). A preclinical canine model for composite tissue transplantation. J. Reconstr. Microsurg.

[16] Erwin Gunanti, Noviana D, Handharyani E (2015). Abstract:Blood, clinical, and histological profile of skin transplants autogenous healing on Indonesian local cat. Proceeding Book of Asian Meeting of Animal Medicine Specialties.

[17] Adams D.C, Ramsey M.L (2005). Grafts in dermatologic surgery. Dermatol. Surg.

[18] Bachmann B.O, Bock F, Weigand S.J, Maruyama K, Dana M.R, Kruse F.E, Leutjen-Drecoll E, Curseifen C (2008). Promotion of graft survival by vascular endothelial growth factor-A neutralization after high risk corneal transplantation. Arch. Opthalmol.

[19] Pressler B.M (2010). Transplantation in small animal. Vet. Clin. N. Am. Small.

[20] Thannoon M.G, Ibrahim S.M, Al-Badrany M.S (2012). Auto-skin transplantation in dog. Bras. J. Vet. Res.

